# Novel treatment strategy with radiofrequency ablation and surgery for pregnant patients with hepatocellular carcinoma: a case report

**DOI:** 10.1186/s40792-018-0434-3

**Published:** 2018-05-02

**Authors:** Megumi Matsuo, Katsunori Furukawa, Hiroaki Shimizu, Hideyuki Yoshitomi, Tsukasa Takayashiki, Satoshi Kuboki, Shigetsugu Takano, Daisuke Suzuki, Nozomu Sakai, Shingo Kagawa, Hiroyuki Nojima, Masayuki Ohsuka

**Affiliations:** The Department of General surgery, Graduate School of Medicine, Chiba University, 1-8-1 Inohana Chuo-ku, Chiba, Japan

**Keywords:** Hepatocellular carcinoma, Pregnancy, Radiofrequency ablation

## Abstract

**Background:**

Hepatocellular carcinoma (HCC) during pregnancy is rare, with a poor prognosis. Recently, however, increasing resection rates have improved survival rate. Currently, various surgeries are safely performed after the second trimester and termination of pregnancy is not always necessary. However, surgery is sometimes limited by gestational age or the patient’s will. When patients with HCC refuse surgery during pregnancy, we face specific problems with respect to curability and fetal life.

Meanwhile, previous studies have revealed radiofrequency ablation (RFA) as a possible alternative to surgery for the treatment of early HCC and shown its favorable local control rate for advanced HCC. However, no case of HCC treated with RFA during pregnancy has yet been reported.

**Case presentation:**

Here, we present the case of a 33-year-old woman, who was a hepatitis B virus carrier. The patient had been followed up because HBV carrier could develop hepatitis or HCC. And she was diagnosed with a 40-mm HCC tumor at 17 weeks of gestation. She refused surgery because she was pregnant and wanted to continue her pregnancy; therefore, we performed RFA for the local control of her HCC at 17 weeks of gestation and radical surgery at postpartum. She delivered a healthy baby and has survived without recurrence for 6 years after the surgery.

**Conclusions:**

Surgery is potentially a curative treatment for HCC whether the patient is pregnant or not. However, various problems unique to pregnancy make it difficult to perform a straightforward surgery. Our case revealed that RFA can be safely performed in pregnant patients during the second trimester, and the combination of RFA and surgery can radically increase the resection rate of HCC during pregnancy.

## Background

Hepatocellular carcinoma (HCC) during pregnancy is extremely rare, with only 61 cases reported worldwide till date [1–11]. Prognosis was believed to be very poor but has recently improved because of the increased rates of radical resection [1, 2]. However, we are confronted with the dilemma of choosing between curability and fetal life regarding treatment options of HCC during pregnancy. Recently, various surgeries including hepatectomy have been safely performed during the second and third trimesters [2], and patients do not always need to terminate their pregnancy. However, some patients refuse surgery while pregnant, even if radical resection is possible, and they want to continue their pregnancy. No alternative treatment strategy has been reported except delaying surgery until delivery in such cases.

In non-pregnant patients with HCC, the literature has revealed radiofrequency ablation (RFA) to be an alternative treatment to surgery for early HCC, with high rates of local control and promising survival rates in advanced HCC [12]. However, there has been no report of RFA for HCC during pregnancy.

Here, we present such a case treated by a combination of RFA and surgery with ensuing 6-year disease-free survival.

## Case presentation

**Fig. 1:**
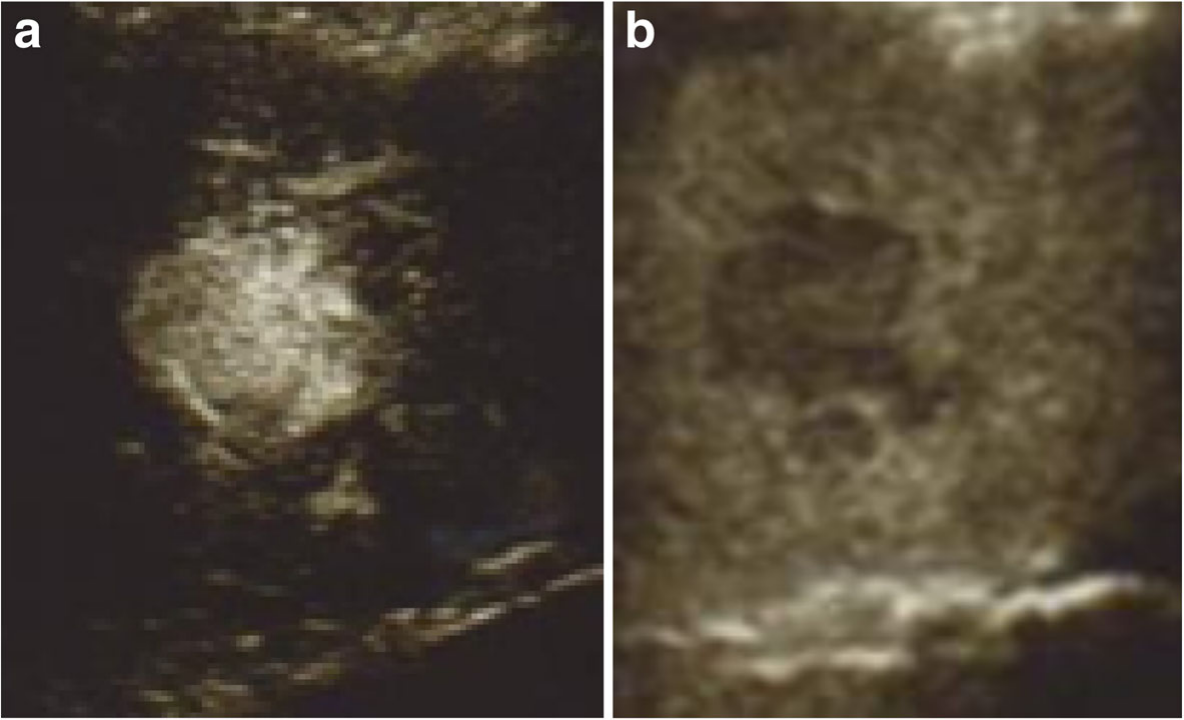
The tumor in segment VIII by enhanced ultrasound sonography at 17 weeks of gestation. **a** Early enhanced 32-mm tumor. **b** Late washed-out tumor

A 33-year-old woman, who was a hepatitis B virus carrier, had been followed up since she was 17 years old because a HBV carrier could develop hepatitis or HCC. Annular ultrasound sonography showed a 24-mm tumor in the liver segment VIII, during which time she was in the eighth week of gestation. Laboratory findings showed an alpha-fetoprotein (AFP) level of 39.2 ng/mL, AFP-L3 (*Lens culinaris* agglutinin-reactive fraction of AFP) of 2.8%, and des-γ-carboxy prothrombin (DCP) level of 468 mAU/mL. We suspected HCC but could not make a definitive diagnosis because enhanced image tests were limited due to the gestation. Contrast-enhanced ultrasound sonography showed a 37-mm tumor with a classical HCC pattern (Fig. 1) at 17 weeks of gestation. We diagnosed it as HCC and recommended resection during her pregnancy, but the patient refused surgery because she was concerned about the negative impact on her fetus, although we explained the safety of the surgery during the second trimester to her. We believed that RFA could be an alternative treatment option during pregnancy, but it was not sufficient to achieve curability because of her HCC size. Therefore, we got an informed consent about the benefits and risks of RFA from the patient and we planned to conduct RFA during pregnancy followed by surgery after the delivery.

**Fig. 2:**
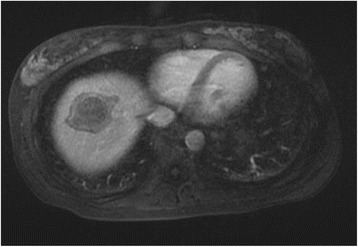
EOB-MRI at 24 weeks of gestation. Non-enhancement of a 40-mm tumor

We performed RFA at 17 weeks of gestation, and any complications did not occur. In a dynamic study, gadoxetic acid-enhanced magnetic resonance (EOB-MRI) imaging showed a non-enhanced 40-mm tumor at 24 weeks of gestation (Fig. 2). She delivered a healthy baby at 37 weeks of gestation.

**Fig. 3:**
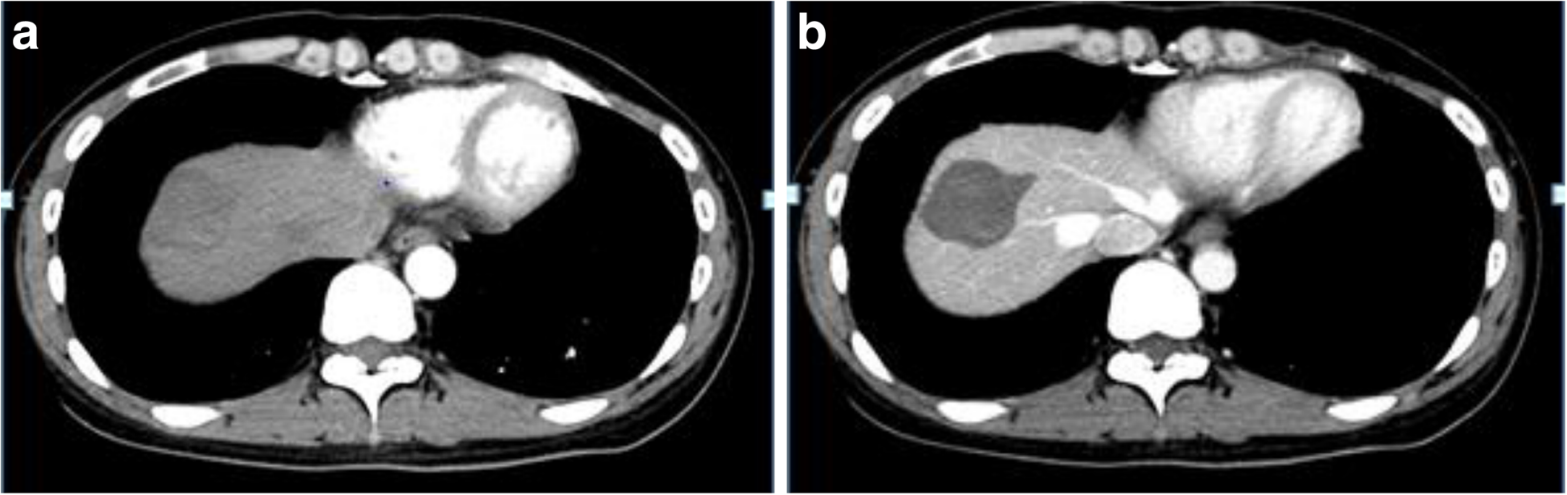
MDCT at 13 days postpartum. **a** Non-enhancement of a tumor in early phase. **b** Non-enhancement of a tumor in late phase

Multi-detector row computed tomography (MDCT) 13 days postpartum showed a 45-mm non-enhanced mass without any other new lesions (Fig. 3). Although there was no proof of residual viable lesion in MDCT, we were skeptical about the curability only by RFA because of the evidence that local recurrence rate increased in RFA for advanced HCC. Therefore, we decided to perform the surgery at postpartum as originally planned under well-informed consent.

**Fig. 4:**
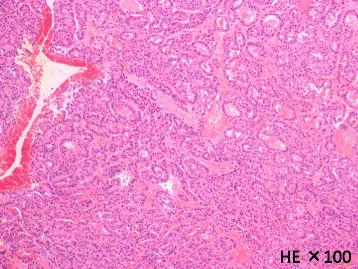
Pathological findings of resected specimen. Moderately differentiated hepatocellular carcinoma, simple nodular type, 40 × 35 mm, thin-trabecular, and pseudoglandular type: eg, fc(+), fc-inf(−), sf(+), s1, vp0, vv0, va0, b0, im(−), sm(−), f2, pT2, N0, M0, and pStageII

**Fig. 5:**
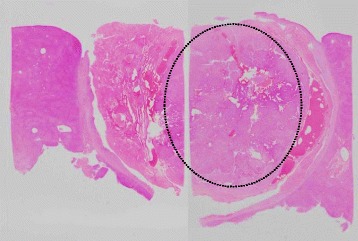
Loupe image of resected specimen. Viable lesions were remained inside of dashed circle

The patient underwent segmentectomy of the liver segment VIII 21 days postpartum, and the postoperative course was uneventful. Pathological evaluation confirmed that RFA resulted in a small viable lesion in necrotic tissue with a free resection margin (Figs. 4 and 5).

She has survived without recurrence for 6 years after surgery, and her child has been healthy as well.

## Discussion

Radical surgery is the best potentially curative therapy for HCC, regardless of pregnancy. Moreover, various surgeries under general anesthesia have been safely performed during the second trimester [13]. Accordingly, termination of pregnancy is not always necessary to perform surgery, except during the first trimester. In addition, delivery before 32 weeks of gestation should be avoided because of fetus immaturity.

In our case, the tumor was resectable at diagnosis but the patient refused to undergo resection because she could not dispel her concern about surgery under pregnancy despite assuring her of the safety. Previous literature reported that the pregnancy itself could promote rapid HCC growth and increase the risk of tumor rupture [14]. Therefore, it could threaten both maternal and fetal life to delay treatment until delivery after 32 weeks of gestation.

We explored an alternative treatment strategy for her and discussed about RFA, the efficacy and the safety for a pregnant patient and her fetus.

A previously published article reported that overall survival and local control of both RFA and surgery for HCC nodules up to 2 cm are the same [12]. In contrast, local recurrence increased in nodules of > 3 cm but local control with RFA was still high [12]. In our case, only RFA could not have attained curability because the nodule was > 4 cm. We expected that RFA could decrease the risk of rapid growth and increase the chance that radical surgery could be performed after delivery.

There are no reports proving the safety of RFA for a pregnant patient. RFA causes tumor necrosis by inducing local hyperthermia accompanied by intravascular thrombosis and microvascular rupture. Because the treatment’s efficacy is localized, limiting complications to the surrounding structures only, we considered RFA might be safe for a patient and her fetus [12].

In addition, we must ponder the possibility of critical complications relating to RFA for pregnant patients such as hemorrhage, symptomatic pleural effusion, or massive ascites although they rarely occur [15].

Regarding other negative influence upon a patient, we must consider about the risk of dissemination by RFA which especially increases in HCC which is poorly differentiated, is highly invasive, and contacts with portal branches [16]. Fortunately, the patient in our case did not have any characters of them and we believed this patient had low risk of dissemination by RFA.

We got the sufficient informed consent about both the benefit and the risks of RFA during pregnancy from the patient and her family and then, we decided to perform RFA for the local control until her delivery. As for medications used during RFA procedure, we usually choose lidocaine for local anesthesia and NSAIDs for pain control in our institution. In this case, we used lidocaine as usual, but we changed NSAIDs into acetaminophen which is safer for the fetus [17].

Pathological analysis revealed most of the tumor had become necrotic after RFA, and this proved to provide favorable local control of HCC. Moreover, the left viable lesion proved that the combination of RFA and surgery could indeed accomplish curability in our case.

Patients in our case have survived without recurrence for 6 years after the surgery. However, a previous article reported that RFA itself increased recurrent risk in a particular type of HCC which shows triple positive tumor marker: AFP, AFP-L3, and DCP [18]. Both AFP and DCP were elevated but not AFP-L3 in our case. The problem is that AFP and AFP-L3 usually elevate due to pregnancy and it is difficult to evaluate the increase of those tumor markers straightforwardly as prognostic factors for pregnant patients with HCC. Even though, we must consider about this aggressive phenotype whenever we plan to perform RFA for HCC regardless of pregnancy.

In our case, no complication related to RFA occurred and RFA could be safely performed in our case; however, this was not enough to prove its safety during the second trimester. Moreover, its safety status during the first trimester is unknown. If RFA could be a choice throughout pregnancy and not present a risk to maternal or fetal life, it may be applicable in more cases. We expect to see more case reports demonstrating the safety and efficacy of RFA for HCC during pregnancy.

## Conclusions

Surgery is the first choice for resectable HCC regardless of pregnancy. However, we must make a treatment decision depending on fetal maturity in HCC during pregnancy. RFA is probably safe for both pregnant patients and their fetus, and it can be an applicable choice for the management of patients with HCC during the second trimester until radical surgery can be accomplished. However, we need further analysis to evaluate the benefit and risk of RFA during pregnancy.

## Abbreviations

AFP: Alpha-fetoprotein
AFP-L3: *Lens culinaris* agglutinin-reactive fraction of AFP
DCP: Des-γ-carboxy prothrombin
EOB-MRI: Gadoxetic acid-enhanced magnetic resonance
HCC: Hepatocellular carcinoma
MDCT: Multi-detector row computed tomography
RFA: Radiofrequency ablation
